# Genotype-Based Severity Scoring System in Wolfram Syndrome: Correlation with Onset of Cardinal Symptoms and WFS1 Gene Variant Types

**DOI:** 10.64898/2026.03.24.26349216

**Published:** 2026-03-26

**Authors:** Liam Oiknine, Abby F. Tang, Fumihiko Urano

**Affiliations:** 1 Department of Medicine, Division of Endocrinology, Metabolism, and Lipid Research, Washington University School of Medicine, 660 South Euclid Avenue, St. Louis, MO 63110, USA; 2 Department of Pathology and Immunology, Washington University School of Medicine, 660 South Euclid Avenue, St. Louis, MO 63110, USA

**Keywords:** Wolfram syndrome, WFS1, genotype-phenotype correlation, severity scoring, diabetes mellitus, optic atrophy, hearing loss, diabetes insipidus

## Abstract

Wolfram syndrome is a rare genetic disorder characterized by antibody-negative early-onset atypical diabetes mellitus, optic nerve atrophy, sensorineural hearing loss, diabetes insipidus (arginine vasopressin deficiency), and progressive neurodegeneration, with significant variability in disease severity. We assessed the accuracy of a genotype-based severity scoring system to predict the onset of cardinal symptoms in Wolfram syndrome. This system is based on the type of WFS1 variants (in-frame or out-of-frame) and their location relative to transmembrane domains. Severity scores were assigned to 324 patients with documented onset ages for diabetes mellitus, optic atrophy, hearing loss, and diabetes insipidus. Our analysis revealed a clear correlation between severity scores and earlier onset of diabetes mellitus and optic atrophy. Patients with in-frame variants outside transmembrane domains exhibited milder symptoms, especially WFS1 c.1672C>T (p.Arg558Cys) variant, whereas those with out-of-frame variants showed the earliest onset. Severity scores 3 and 4 did not follow the expected progression, suggesting that transmembrane domain involvement in both alleles may result in greater severity. These findings suggest that this scoring system provides valuable insights into the progression of Wolfram syndrome and may guide clinical care. Further refinement may improve its utility for predicting the onset of non-diabetic symptoms.

## 1. Introduction

Wolfram syndrome is a rare autosomal recessive disorder characterized by early-onset antibody-negative diabetes mellitus, optic nerve atrophy, sensorineural hearing loss, diabetes insipidus (arginine vasopressin deficiency) and progressive neurodegeneration [1; 2]. Most patients carry biallelic pathogenic variants in the *WFS1* gene, whereas a smaller subset carry biallelic pathogenic variants in the *CISD2* gene [3]. The objective of this study is to assess the accuracy of our current categorization system for the severity of Wolfram syndrome [4]. Severity is defined by the age of onset of the core manifestations of Wolfram syndrome, with particular focus on diabetes mellitus, which appears to be a key indicator for overall disease severity. The system is based on two features demonstrated to significantly impact the severity of disease manifestations. First, the type of pathogenic variants in the WFS1 gene has been shown to influence the severity of Wolfram syndrome. Specifically, patients with in-frame missense or insertion/deletion pathogenic variants tend to exhibit milder manifestations than those with frameshift/nonsense pathogenic variants, as evidenced by later onset ages for diabetes mellitus and optic atrophy. Secondly, in-frame variants located within transmembrane domains result in more severe manifestations compared to those outside transmembrane domains.

## 2. Materials and Methods

### Patients

Subjects, and their parents or legal guardians, as appropriate, provided written, informed consent before participating in this study, which was approved by the Human Research Protection Office at Washington University School of Medicine in St. Louis, MO (IRB ID #201107067). Patient data from the Washington University International Registry and Clinical Study for Wolfram Syndrome and patient case reports were analyzed to select for patients with two recessive pathogenic variants in the WFS1 gene. Patients were excluded if they lacked genetic information for either of their WFS1 allele variants. Additionally, records were excluded if they did not have a numerical age of onset for their respective clinical phenotype (diabetes insipidus, optic atrophy, diabetes mellitus, hearing loss). Pathogenic variants were then classified as being either nonsense/frameshift variants or missense/in-frame insertion and deletion variants.

### Data Analysis

The data used for this analysis was gathered from a sample of more than 400 Wolfram patients, with varying amounts of clinical data. In total, onset information was present for 324, 306, 195, and 149 patients for Diabetes Mellitus, Optic Atrophy, Hearing Loss, and Diabetes Insipidus respectively. Patients were divided into subgroups based on the symptoms they had onset ages listed for, and each group was graphed independently excluding outliers. Statistical significance between groups was analyzed using the Kruskal-Wallis test and Dunn’s test with Bonferroni correction. Statistical significance was defined by a p-value < 0.05.

## 3. Results

**Figure 1.**
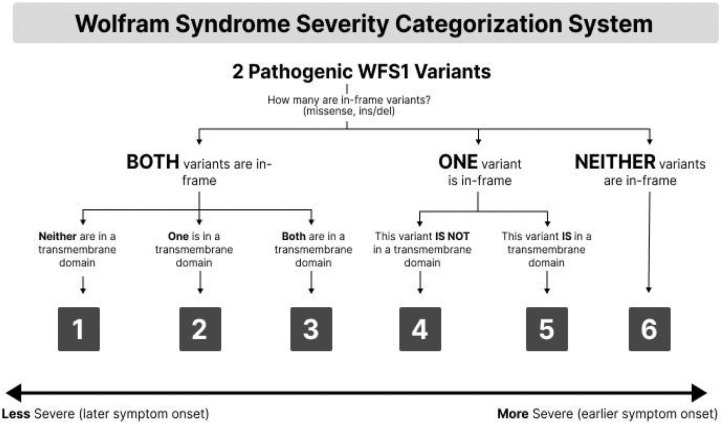
Severity scoring system schematic illustrating the six genotype-based groups ordered by increasing disease severity.

When the type of WFS1 variants and their location relative to transmembrane domains are considered together [5], they form six distinct groups of patients, ordered by increasing disease severity. The severity scores are defined as follows (Figure 1):

- **Severity Score 1:** Both alleles contain in-frame (missense, ins/del) variants; neither variant lies within a transmembrane domain.
- **Severity Score 2:** Both alleles contain in-frame (missense, ins/del) variants; one variant lies within a transmembrane domain, the other does not.
- **Severity Score 3:** Both alleles contain in-frame (missense, ins/del) variants; both variants lie within transmembrane domains.
- **Severity Score 4:** One allele contains an in-frame (missense, ins/del) variant, the other contains an out-of-frame (frameshift/nonsense) variant. The in-frame variant is not located in a transmembrane domain.
- **Severity Score 5:** One allele contains an in-frame (missense, ins/del) variant, the other contains an out-of-frame (frameshift/nonsense) variant. The in-frame variant is located in a transmembrane domain.
- **Severity Score 6:** Both alleles contain out-of-frame (frameshift/nonsense) variants.

### Diabetes Mellitus

**Figure 2.**
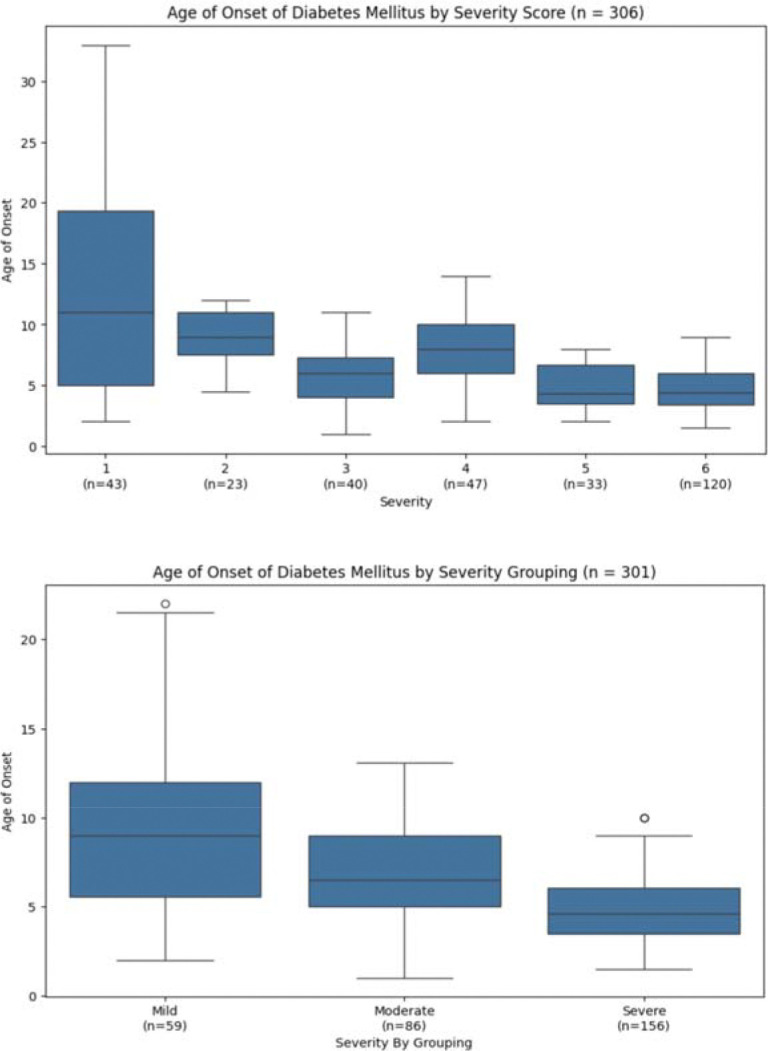
Diabetes Mellitus onset age by individual severity score (top) and by consolidated Mild/Moderate/Severe groups (bottom). Statistical significance between scores 2 and 3 (p = 0.008128) and scores 4 and 5 (p = 0.001535). Consolidated group comparisons: Mild vs. Moderate p = 0.011445; Moderate vs. Severe p = 5.25 × 10^−6^. Median onset ages: 9.0, 6.5, and 4.6 years for ‘Mild’, ‘Moderate’, and ‘Severe’ respectively.

Diabetes mellitus follows a clear trend with earlier ages of onset correlating to higher severity scores, the only exception being severity score 3. Statistical significance was seen between scores 2 and 3 (p = 0.008128) as well as scores 4 and 5 (p = 0.001535), serving as strong breaking points for consolidating the data into three groups (Figure 2, upper panel, Table 1, Table 2). The ‘Mild’ group consists of severity scores 1–2, the ‘Moderate’ group consists of severity scores 3–4, and the ‘Severe’ group consists of severity scores 5–6. Consolidating these groups allowed for statistical significance to be achieved between each of the three groups (Mild vs. Moderate: p = 0.011445; Moderate vs. Severe: p = 5.25 × 10^−6^). These groups, with median onset ages of 9.0, 6.5, and 4.6 years for ‘Mild,’ ‘Moderate,’ and ‘Severe’ respectively, may serve as accurate benchmarks for predicting DM onset ages based on genotype (Figure 2, **lower panel**).

### Optic Atrophy

**Figure 3.**
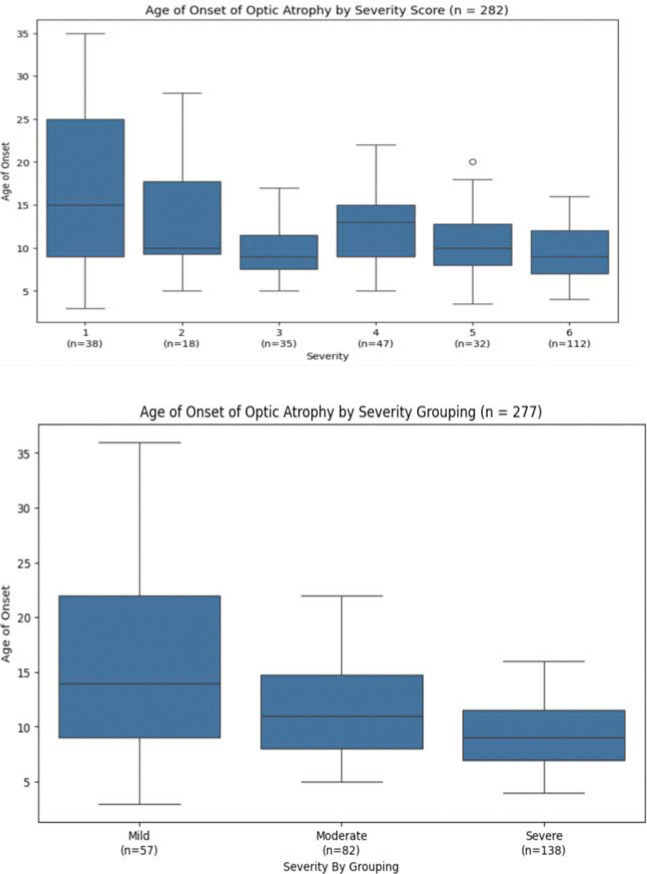
Optic Atrophy onset age by individual severity score (top) and by consolidated Mild/Moderate/Severe groups (bottom). Near statistical significance between groups 3 and 4 (p = 0.059694). Median onset ages: 14.0, 11.0, and 9.0 years for ‘Mild’, ‘Moderate’, and ‘Severe’ respectively.

Optic atrophy follows a somewhat linear trend, though less consistently than diabetes mellitus. Group 3 again shows earlier onset compared to group 4. Near statistical significance was observed between adjacent groups 3 and 4 (p = 0.059694), but no two adjacent groups reached significance (Figure 3, upper panel, Table 1, Table 2). The data was consolidated into three subgroups (‘Mild’, ‘Moderate’, and ‘Severe’) according to the same breakdown of severity scores as defined above, with median onset ages of 14.0, 11.0, and 9.0 years respectively (Figure 3, **lower panel**). This representation shows a clearer trendline correlating higher severity scores to earlier onset of optic atrophy, though these groups are less clearly defined compared to those of diabetes mellitus. A potential contributing factor is that optic atrophy may only be noticed gradually after onset, potentially introducing reporting bias in the recorded onset ages.

### Hearing Loss and Diabetes Insipidus

**Figure 4.**
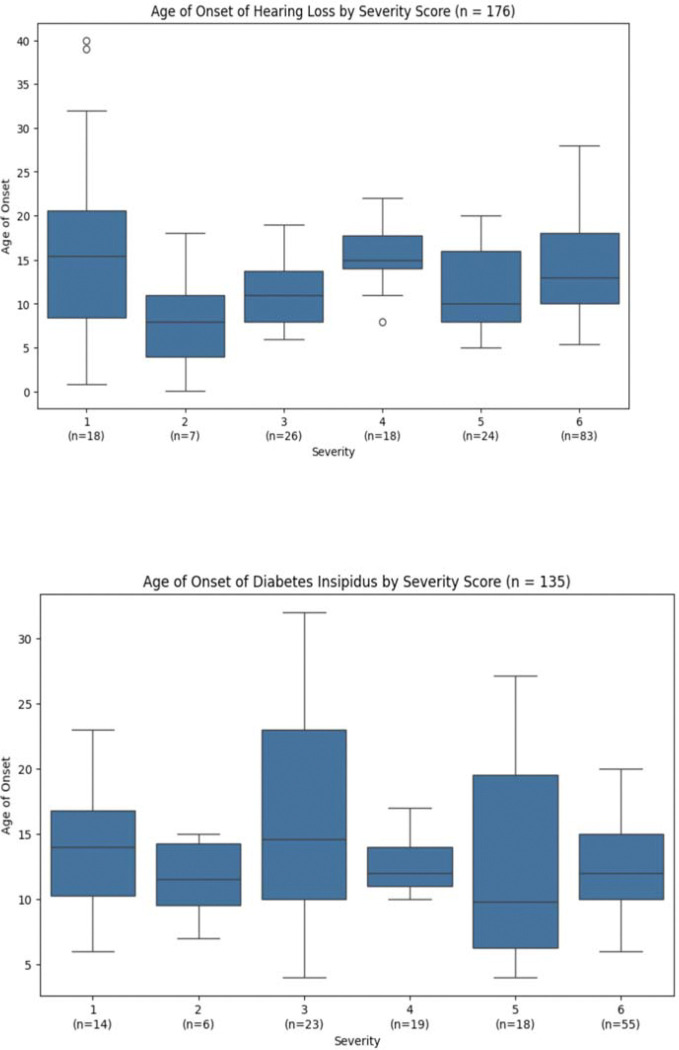
Hearing Loss (top) and Diabetes Insipidus (bottom) onset age by severity score. No statistical significance between any adjacent groups and no consistent pattern is apparent.

Hearing loss and diabetes insipidus data do not follow any specific pattern, indicating no correlation between age of onset and the current severity scoring system. This is consistent with prior studies showing that neither variant type nor transmembrane domain location significantly influences these manifestations. Notably, both hearing loss and diabetes insipidus graphs share similar shapes, with earlier onset ages observed in patients with scores 2 and 5 (Figure 4, Table 1, Table 2).

## 4. Discussion

Our scoring system demonstrates consistency for diabetes mellitus and optic atrophy, and shows no significant correlation with age of onset for hearing loss or diabetes insipidus. While the system provides prognostic insight for these two cardinal manifestations, further refinement is needed to improve its predictive accuracy and broaden its clinical utility.

Several avenues may guide this refinement. Wolfram syndrome is considered a prototype endoplasmic reticulum (ER) disease [6; 7], and functional assays quantifying ER stress levels associated with individual WFS1 variants could offer a more granular and biologically grounded basis for severity classification [8]. In addition, circulating biomarkers measured in our patients, including C-peptide and neurofilament light chain, as well as emerging ER stress markers such as mesencephalic astrocyte neurotrophic factor, may help stratify disease severity more precisely and refine score boundaries [9; 10; 11]. Structural modeling of each variant using tools such as AlphaFold may similarly help predict functional impact, including effects on protein stability, membrane insertion, and inter-domain interactions, beyond what variant type and transmembrane domain location alone can capture [12; 13]. Finally, continued patient recruitment to our international registry (https://wolframsyndrome.wustl.edu/), along with systematic collection of genetic and longitudinal clinical data, will expand the sample sizes needed to draw more robust conclusions, particularly for hearing loss and diabetes insipidus, where our current cohort is likely underpowered to detect meaningful genotype-phenotype relationships.

A notable anomaly within the scoring framework is that severity score 3, defined by two in-frame variants both located within transmembrane domains, appears more severe than score 4, which combines one frameshift or nonsense variant with one in-frame variant outside a transmembrane domain. This is counterintuitive given that frameshift and nonsense variants are generally expected to produce greater loss of WFS1 function than in-frame variants. One possible explanation is that simultaneous disruption of transmembrane domains on both alleles impairs protein folding and membrane topology more severely than a single truncating variant paired with a partially functional allele, perhaps because residual WFS1 activity from the milder allele in score 4 patients is sufficient to provide a degree of functional compensation. Consistent with this interpretation, severity score 3 is associated with earlier median onset ages for diabetes mellitus, optic atrophy, and hearing loss relative to score 4. Repositioning scores 3 and 4 in the rating system may therefore improve its face validity, though this reordering has limited practical consequences under the consolidated three-tier grouping, in which both scores fall within the ‘Moderate’ category, which performs robustly despite this internal discrepancy.

Given that hearing loss and diabetes insipidus show no correlation with the current rating scale, it is possible that the genetic and molecular determinants of these manifestations may be distinct from those driving diabetes mellitus and optic atrophy onset. Future work aimed at identifying variant-specific effects on auditory hair cell function and arginine vasopressin-producing neurons may ultimately support the development of separate, manifestation-specific severity scales that better capture the full phenotypic complexity of Wolfram syndrome.

This study provides a systematic evaluation of a genotype-based severity scoring system in Wolfram syndrome, applied to one of the largest patient cohorts examined to date. The results demonstrate that WFS1 variant type and transmembrane domain involvement are useful predictors of diabetes mellitus and optic atrophy onset, and that a consolidated three-tier severity classification, comprising Mild, Moderate, and Severe groups, potentially offers a clinically practical framework for prognostic counseling. These findings represent a step toward personalized medicine in Wolfram syndrome, where early and accurate prediction of disease trajectory could inform the timing of clinical monitoring, guide enrollment in interventional trials, and support family counseling at the time of diagnosis. As therapeutic strategies targeting ER stress and WFS1 function continue to advance [14; 15], a well-validated severity scoring system will be an increasingly valuable tool for patient stratification and outcome assessment. We anticipate that integration of functional, structural, and biomarker data into future iterations of this system will substantially improve its scope and predictive power across all major manifestations of this devastating disease.

## Figures and Tables

**Table 1.** Median Age of Onset – Full Dataset

	Diabetes Mellitus	Optic Atrophy	Hearing Loss	Diabetes Insipidus
Full Dataset (n)	324	306	195	149
Median Onset Age (years)	6.0	11.0	14.0	13.0

**Table 2.** Median and Mean Age of Onset by Severity Score

Severity Score	Median Age of Onset (years)				Mean Age of Onset (years)			
	DM	OA	HL	DI	DM	OA	HL	DI
1	11.0	15.0	15.5	14.0	13.0	16.9	16.4	13.3
2	9.0	10.0	8.0	11.5	9.0	13.2	8.0	11.5
3	6.0	9.0	11.0	14.6	5.9	9.8	11.1	16.4
4	8.0	13.0	15.0	12.0	7.8	12.7	15.5	12.6
5	4.3	10.0	10.0	9.8	4.8	10.6	11.7	13.0
6	4.4	9.0	13.0	12.0	4.9	9.5	14.3	12.4

DM = Diabetes Mellitus; OA = Optic Atrophy; HL = Hearing Loss; DI = Diabetes Insipidus

## Data Availability

The clinical data supporting the findings of this study are not publicly available due to patient privacy considerations and restrictions of the IRB-approved protocols governing the Washington University International Registry and Clinical Study for Wolfram Syndrome (IRB #201107067). Deidentified data may be made available from the corresponding author, Fumihiko Urano, MD, PhD (urano@wustl.edu), upon reasonable request and subject to institutional review and execution of an appropriate data use agreement.
